# Supplementary material for: Effects of long-term in vivo micro-CT imaging on hallmarks of osteopenia and frailty in aging mice
Source: PLoS One. 2020 Sep 23;15(9):e0239534. doi: 10.1371/journal.pone.0239534 (PMC7511008; doi:10.1371/journal.pone.0239534)
Supplement: S1 Table — The p-values and effect sizes (f) of the main and interaction effects, respectively are listed below (significance level α = 0.05). (DOCX) [file pone.0239534.s001.docx]

**Table S1. The effects of imaging session number (group), genotype and the interaction effect (genotype*group) on bone morphometric parameters and frailty index (FI) at 40 weeks of age were compared via two-way ANOVA analysis.** The p-values and effect sizes (*f*) of the main and interaction effects, respectively are listed below (significance level α =0.05).

| two-way ANOVA | **Interaction** | | **Group** | | **Genotype** | |
| --- | --- | --- | --- | --- | --- | --- |
|  | p value | effect size *f* | p value | effect size *f* | p value | effect size *f* |
| **BV/TV** | 0.081 | 0.31 | <0.0001 | 0.74 | 0.001 | 0.50 |
| **Tb.Th** | 0.061 | 0.33 | 0.005 | 0.46 | <0.0001 | 0.57 |
| **Ct.Ar/Tt.Ar** | 0.200 | 0.25 | 0.004 | 0.48 | <0.0001 | 0.84 |
| **Ct.Th** | 0.279 | 0.22 | 0.049 | 0.34 | <0.0001 | 0.86 |
| **BFR** | 0.475 | 0.17 | 0.008 | 0.44 | 0.005 | 0.39 |
| **BRR** | 0.411 | 0.28 | <0.05 | 0.44 | <0.0001 | 0.59 |
| **MAR** | 0.403 | 0.18 | 0.065 | 0.32 | 0.029 | 0.30 |
| **MRR** | 0.641 | 0.13 | 0.002 | 0.50 | <0.0001 | 1.21 |
| **FI** | 0.143 | 0.27 | 0.054 | 0.33 | <0.0001 | 1.09 |
