# Supplementary material for: Effects of long-term in vivo micro-CT imaging on hallmarks of osteopenia and frailty in aging mice
Source: PLoS One. 2020 Sep 23;15(9):e0239534. doi: 10.1371/journal.pone.0239534 (PMC7511008; doi:10.1371/journal.pone.0239534)
Supplement: S2 Table — (Significance level α = 0.05). (DOCX) [file pone.0239534.s002.docx]

**Table S2. P-values, effect sizes (*f*) and achieved power obtained by cross-sectional (one-way ANOVA) and longitudinal analysis (paired t-test)**. (significance level α =0.05).

| **Cross-sectional analysis** | one-way ANOVA | **WT** | | | **PolgA** | | |
| --- | --- | --- | --- | --- | --- | --- | --- |
|  |  | p-value | effect size *f* | achieved power | p-value | effect size *f* | achieved power |
|  | **BV/TV** | 0.007 | 0.71 | 0.88 | <0.0001 | 0.96 | 0.99 |
|  | **Tb.Th** | 0.087 | 0.48 | 0.53 | 0.001 | 0.75 | 0.97 |
|  | **Ct.Ar/Tt.Ar** | 0.030 | 0.58 | 0.72 | 0.088 | 0.41 | 0.52 |
|  | **Ct.Th** | 0.125 | 0.43 | 0.46 | 0.146 | 0.36 | 0.42 |
|  | **BFR** | 0.285 | 0.33 | 0.26 | 0.020 | 0.54 | 0.66 |
|  | **BRR** | 0.151 | 0.41 | 0.42 | 0.023 | 0.53 | 0.64 |
|  | **MAR** | 0.687 | 0.18 | 0.11 | 0.054 | 0.45 | 0.61 |
|  | **MRR** | 0.033 | 0.57 | 0.70 | 0.044 | 0.47 | 0.53 |
| **Longitudinal analysis** | paired t-test | **WT** | | | **PolgA** | | |
|  |  | p-value | effect size *f* | achieved power | p-value | effect size *f* | achieved power |
|  | **BV/TV** | <0.0001 | 0.52 | 0.99 | 0.005 | 0.45 | 0.99 |
|  | **Tb.Th** | <0.0001 | 0.86 | 1.00 | 0.014 | 0.39 | 0.96 |
|  | **Ct.Ar/Tt.Ar** | <0.0001 | 0.57 | 1.00 | 0.011 | 0.29 | 0.80 |
|  | **Ct.Th** | <0.0001 | 0.91 | 1.00 | 0.002 | 0.36 | 0.92 |
|  | **BFR** | 0.050 | 0.59 | 1.00 | 0.094 | 0.32 | 0.85 |
|  | **BRR** | 0.091 | 0.43 | 0.93 | 0.314 | 0.22 | 0.56 |
|  | **MAR** | 0.698 | 0.11 | 0.15 | 0.988 | 0.01 | 0.05 |
|  | **MRR** | 0.019 | 0.52 | 0.99 | 0.610 | 0.10 | 0.15 |
